# A pilot study on children with bone-age advancement without early sexual maturation; auxological and laboratory (GNRH stimulation) characteristics

**DOI:** 10.1186/1687-9856-2013-S1-O29

**Published:** 2013-10-03

**Authors:** Choi Byung Ho, Park Hyo Min, Lee Sang In, Ko Cheol Woo

**Affiliations:** 1 Kyungpook National University Hospital, Daegu, Korea

## 

Social concern over precocious puberty (PP) is rising nowadays, and there is actually increased visit to hospital due to PP. Among them children with no pubertal sign showing advanced bone-age (BA) have been found in many cases. This study was done to see clinical and laboratory characteristics in these children with so called 'asymptomatic PP'.

Among children whom visited for evaluation of early sexual maturation between July 2007 and June 2009, children with advanced BA more than 1 yr without any pubertal sign were enrolled. Their clinical, laboratory data including GnRH stimulation test and auxological data were analyzed retrospectively.

Thirty-eight children with asympomatic PP were enrolled. Male:female ratio was 1:1. Chronological ages (CA) were 9.3±1.1 yrs in boys, 8.1±1.4 yrs, in girls. BA were 11.3±0.9 yrs in boys, 9.8±1.4 yrs in girls. Positive result (peak LH>5 IU/L) of GnRH stimulation test was found in 18 out of 38 children (47.4%) -18 boys (69%) and 8 girls (31%). Patients were divided into 2 groups (positive and negativ e group) based on GnRH stimulation test. Basal LH value of positive group was significantly higher than negative group in boys (0.4±0.5 vs 0.1±0.1 IU/L, respectively, p<0.05) and girls (1.0±1.7 vs 0.1±0.0 IU/L, respectively, p<0.05). However, there were no significant differences between groups in height, chronological age, progression of BA, BMI.

Children with BA advancement without secondary sexual characteristics was found. Their clinical course is unknown yet. Further study is necessary to confirm chracteristics of BA advancement without early sexual maturation, so-called 'asymptomatic PP'.

